# Pulmonary Nocardiosis in an Immunocompetent Patient with Cystic Fibrosis

**DOI:** 10.1155/2015/984171

**Published:** 2015-04-15

**Authors:** Lucy Schoen, Jonathan D. Santoro, Carlos Milla, Sumit Bhargava

**Affiliations:** ^1^Stanford University School of Medicine, 291 Campus Drive, Li Ka Shing Building, Stanford, CA 94305-5101, USA; ^2^Department of Pediatrics, Stanford Children's Hospital, 725 Welch Road, Palo Alto, CA 94304, USA; ^3^Division of Pulmonary Medicine, Department of Pediatrics, Stanford Children's Hospital, 725 Welch Road, Palo Alto, CA 94304, USA

## Abstract

*Nocardia* spp. are bacteria of low virulence that cause infection classically in immunocompromised hosts with the lungs as the primary site of infection in the majority of cases. Patients with cystic fibrosis have pulmonary disease characterized by frequent and progressive bacterial infections. Reports of *Nocardia* spp. isolation in CF are rare in the literature and may represent colonization or active infection, the significance and optimal treatment of which are unknown. We report the second case to date of *Nocardia transvalensis* pulmonary infection in an immunocompetent patient with CF and the first in a child under the age of eighteen.

## 1. Background

*Nocardia* spp. are gram positive, intracellular bacteria of low virulence found worldwide in soil and most frequently cause opportunistic infection in immunocompromised hosts. Incidence has been estimated at 1,000 cases annually in the United States, although rates are increasing [1]. Risk factors for nocardiosis include glucocorticoid use, IV drug use, previous transplant, acquired immune deficiency syndrome, and underlying pulmonary disease including chronic obstructive pulmonary disease, pulmonary fibrosis, and silicosis [2]. Immunocompetent hosts represent 10–50% of cases of infection [3]. Disease spectrum varies and includes pulmonary, neurologic, and/or cutaneous involvement. The lungs are the primary site of infection in two-thirds of cases, with radiographic findings ranging from small nodules to bilateral infiltrates with cavitation [3].

Cystic fibrosis (CF) is an autosomal recessive disease caused by mutations in a chloride ion channel resulting in multiple organ involvement with pulmonary manifestations as the major contributor to morbidity and mortality. Viscous secretions and chronic obstruction lead to colonization by bacteria and acute and chronic infection. Bacterial species influence progression of irreversible lung disease and greatly affect morbidity and mortality in this patient population despite their immunocompetence. Initial colonizers include* Staphylococcus aureus* and* Haemophilus influenzae* with* Pseudomonas aeruginosa* presenting later in the disease course [4]. Reports of isolating* Nocardia* spp. on sputum cultures in CF are rare in the United States, begging the question of whether the frequent and aggressive antimicrobial use in CF limits extensive growth of this microorganism [5–9].

The interface between CF and* Nocardia* spp. is fraught with difficulty.* Nocardia* spp. are slow growing and elusive on culture media. Further complicating assessment is a lack of understanding of this organisms' virulence and its role in the microbiota of the lung in a patient with CF. The majority of previously reported cases of* Nocardia* spp. appear to represent colonization of unknown clinical significance rather than active infection [5–8]. Pulmonary* Nocardia* spp. infection in CF has been reported rarely and, as such, the prognosis and optimal treatment are not well defined [7, 10–12]. Here we present a case of* Nocardia transvalensis* infection in an immunocompetent child with CF.

## 2. Case Report

A 9-year-old female with CF (homozygous Q890X mutation) was admitted from an outside hospital for fatigue, cough, and fevers (Tmax 40°C). At baseline her CF-related pulmonary disease was well controlled with a mean forced expiratory volume in one second (FEV1) of 93% when being healthy. She had had recent hospitalization for CF exacerbation with sputum cultures positive for methicillin-resistant* Staphylococcus aureus* and* Pseudomonas aeruginosa *(Table 1). Past medical history was positive for pancreatic insufficiency and chronic allergic bronchopulmonary aspergillosis (ABPA), which was well controlled after prednisolone course two months previously. She lived on a ranch with several domesticated animals but did not perform field work concerning* Nocardia* exposure.

**Figure 1:**
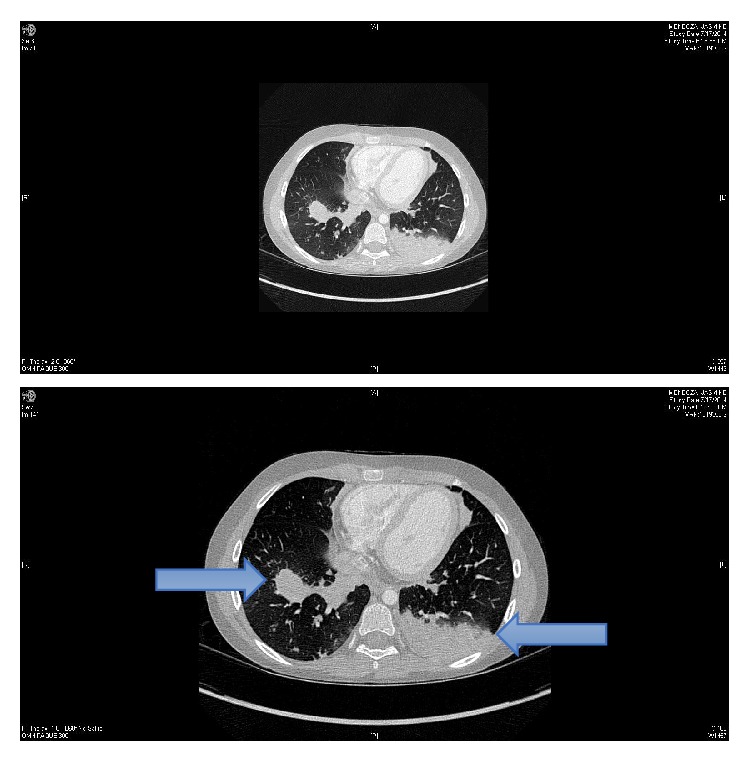
Chest CT, axial view with multifocal pneumonia.

On admission she was continued on ceftriaxone and her outpatient regimen including inhaled tobramycin, itraconazole, azithromycin, dornase alpha, acetylcysteine, and bronchodilators with vest treatments. Lab work including CBC, liver function tests, and chemistries was unremarkable and adjusted neutrophil count was within normal limits. On hospital day one she developed hypoxia and tachypnea requiring supplemental oxygen. Physical exam revealed a young girl in mild respiratory distress with reduced right-sided breath sounds and coarse crackles throughout the lung fields bilaterally without wheezes or stridor. She had a nonproductive cough and full inspiration was limited by right-sided flank pain. The remainder of her exam was unremarkable. Chest radiograph demonstrated a new right lower lobe consolidation. She was started on vancomycin, but despite broadened coverage she continued to be febrile to 40.4°C and complained of pleuritic chest pain. On hospital days one and two her oxygen requirements increased. Chest CT demonstrated a multifocal pneumonia (Figure 1). Initial sputum cultures returned positive for* Nocardia* species and blood cultures were negative. No other organisms were isolated from these cultures. Given continued symptoms despite broad antibiotic coverage and imaging results, the* Nocardia* spp. were considered to be active infection rather than colonization.

Antibiotic coverage was transitioned to IV sulfamethoxazole/trimethoprim (15 mg/kg/day) and within 24 hours fevers resolved, respiratory status improved, and she was weaned off supplemental oxygen. Due to high rates of antibiotic resistance in some* Nocardia* species, double antibiotic coverage was proposed. IV linezolid (30 mg/kg/day) was initiated given favorable susceptibility profiles in* Nocardia* spp. Physical exam revealed no evidence of cutaneous involvement and brain MRI ruled out CNS nocardiosis. Speciation data returned* Nocardia transvalensis* with susceptibilities to amoxicillin-clavulanic acid, linezolid, sulfamethoxazole/trimethoprim, and resistance to amikacin. She tolerated 14 days of IV linezolid and 19 days of IV sulfamethoxazole/trimethoprim with slow improvement in energy and respiratory status. She had severe nausea in the first week of treatment leading to a weight loss of 3 kg (8% body weight). Repeat sputum cultures were negative for* Nocardia* spp. Pulmonary function was reduced compared to baseline with an FEV1 nadir of 50%. She was discharged home on two weeks of oral sulfamethoxazole/trimethoprim and amoxicillin-clavulanic acid (30 mg/kg/day) followed by monotherapy with oral sulfamethoxazole/trimethoprim for a three-month course. Sulfamethoxazole/trimethoprim dose was reduced to 11 mg/kg/day given nausea during hospitalization.

Repeat cultures at one month remained negative for* Nocardia* spp. and FEV1 was improved to 76%. Chest radiograph at two months showed continued opacities and FEV1 remained below baseline at 85%. She underwent bronchoscopy for persistent pulmonary findings and cultures were again negative for* Nocardia* spp. She completed three-month course of sulfamethoxazole/trimethoprim at which time her pulmonary function had returned to baseline with FEV1 of 95%. Work-up for immunodeficiency was negative and chest CT at 6 months displayed no intrapulmonary changes.

## 3. Discussion

Pediatric patients with CF presenting with* Nocardia* spp. infection are rare but recent reports by Rodriguez-Nava et al. and Thorn et al. indicate a growing awareness that challenges prior data indicating* Nocardia* spp. as a pathogen of immunocompromised adults [3, 7, 8]. These larger scale studies present patients with both colonization and active infection, which can be difficult to differentiate given underlying lung pathology in CF and frequent growth of multiple pathogens on sputum culture causing debate with regard to clinical need to treat [7]. The authors of these studies advocate therapy when symptoms are present and* Nocardia* spp. are isolated alone or when symptoms persist in the context of multiple pathogens despite broad antibiotic coverage.

The standard treatment for pulmonary nocardiosis includes sulfamethoxazole/trimethoprim, although there have been reported cases of resistance [3, 7]. Treatment duration is usually 3–6 months but is not standardized and varies based on clinical course and comorbidities [3]. In CF patients with reported* Nocardia* spp. infections, antibiotic durations ranged from 15 days to over 6 months. The majority of patients received sulfamethoxazole/trimethoprim alone or in combination with other agents including amoxicillin-clavulanic acid, clarithromycin, ciprofloxacin, ceftazidime, or amikacin [7, 10–12]. Therapy with linezolid alone has also been used [6]. The only prior report of* Nocardia transvalensis* infection in a patient with CF was successfully treated with two weeks of IV sulfamethoxazole/trimethoprim and linezolid followed by one week of oral linezolid and four weeks of oral sulfamethoxazole/trimethoprim [10].

Our case report presents the second report of* Nocardia transvalensis* in a patient with CF in the United States and the first in a child under 18. This subspecies of* Nocardia* has been previously identified as more virulent in mice than the more prevalent* Nocardia asteroides* subspecies [13]. Patients are more likely to develop greater symptom burden with this subspecies but general susceptibility patterns are favorable with common resistance only to amikacin noted at 18% [14]. Morbidity and mortality associated with this microorganism are generally limited to dissemination from the pulmonary system which is rare in immunocompetent patients. Further, assessment of dissemination can be nearly impossible to discern as growth on blood culture is almost universally negative [13]. Of note, there has been one case report in adult literature describing pulmonary nocardiosis with the presence of ABPA, which as with our patient may indicate need to treat [15]. Oral corticosteroids have been previously proposed as a risk factor for development of nocardiosis [3]; however, it is unknown whether ABPA or its therapy is a risk factor for* Nocardial* colonization or infection in CF.

The effect of* Nocardia* spp. and specifically* Nocardia transvalensis* colonization of the lung tissue is unknown, making it difficult to ascertain whether asymptomatic patients warrant antimicrobial therapy. Microbial burden in patients with CF has been studied previously, demonstrating need for eradication of certain microorganisms, but this has not been established for* Nocardia* spp. [16]. Previous studies have demonstrated an increased severity of symptoms with concurrent* Nocardia* spp. and* Pseudomonas* spp. infections, although this observation is strictly clinical in nature [17].

The microbiome of the CF patient's pulmonary system is diverse and fragile. While we advocate for antimicrobial stewardship, we believe that treatment of* Nocardia transvalensis* in patients with CF is necessary as both our patient and that of the previous reported case presented with active infection. The association between greater symptom burden and coinfection with* Pseudomonas* spp. in conjunction with the greater virulence of this* Nocardia* subspecies is enough to warrant therapy, especially in patients with CF who cannot easily tolerate pulmonary insults. Additionally based on a literature review and the results of our study and Aravantagi et al., we advocate for a three-month course of sulfamethoxazole/trimethoprim (initially by IV route then PO) in conjunction with IV linezolid during acute hospitalization [10].

This case is unique in both its rarity and complexity. We seek to highlight our case and recent literature in the setting of increased reports of* Nocardia* spp. colonization and infection in immunocompetent patients with CF over the last 20 years [18].

## 4. Conclusion

Our report seeks to stress the importance of antimicrobial therapy in patients with CF presenting with* Nocardia transvalensis* infection as the virulence of this organism appears to be greater than other* Nocardia* species in patients with CF. Finally we also would like to purpose a possible correlation between a diagnosis of ABPA in CF and developing* Nocardia* spp. infection.

## Figures and Tables

**Table 1a:** (a) *Pseudomonas aeruginosa* (nonmucoid CF)

Antibiotic	Sensitivity
Amikacin	Susceptible
Method: *KB *	
Aztreonam	Susceptible
Method: *KB *	
Ceftazidime	Susceptible
Method: *KB *	
Ciprofloxacin	Susceptible
Method: *KB *	
Meropenem	Susceptible
Method: *KB *	
Piperacillin	Susceptible
Method: *KB *	
Ticarcillin	Intermediate
Method: *KB *	
Tobramycin	Susceptible
Method: *KB *	

**Table 1b:** (b) *Staphylococcus aureus *

Antibiotic	Sensitivity	Microscan
Ciprofloxacin	Resistant	>2
Method: *MIC *		
Clindamycin	Resistant	>4
Method: *MIC *		
Erythromycin	Resistant	>4
Method: *MIC *		
Gentamicin	Susceptible	≤1
Method: *MIC *		
Levofloxacin	Resistant	>4
Method: *MIC *		
Moxifloxacin	Resistant	4
Method: *MIC *		
Oxacillin	Susceptible	
Method: *MIC *		
Penicillin	Resistant	2
Method: *MIC *		
Tetracycline	Resistant	>8
Method: *MIC *		
Trimeth-Sulfamethox	Susceptible	≤0.5/9.5
Method: *MIC *		
Vancomycin	Susceptible	1
Method: *MIC *		
